# Implementation of comprehensive genome analysis in clinical sequencing at an academic institution

**DOI:** 10.1038/s10038-026-01482-x

**Published:** 2026-05-29

**Authors:** Naoko Saito-Sato, Masaki Tanaka, Junko Nomoto, Kanako Koike Fukushima, Masakazu Nishigaki, Shoji Tsuji

**Affiliations:** 1 Center for Genetic Diagnosis, International University of Health and Welfare Narita hospital, Narita-shi, Japan; 2 Institute of Medical Genomics, International University of Health and Welfare, Chiba, Japan; 3 Department of Genetic Counseling, International University of Health and Welfare Graduate School, 4-1-26 Akasaka, Minato, Tokyo, Japan; 4 Present Address: Department of Neurology, National Center Hospital, National Center of Neurology and Psychiatry, 4-1-1 Ogawa-higashi-cho, Tokyo, Japan

**Keywords:** Next-generation sequencing, DNA sequencing, Genetic testing, Sequence annotation, Genetic counselling

## Abstract

Given the increasing number of diseases for which causative genes have been identified, we are facing the need for implementing comprehensive genome sequence analysis as a molecular diagnostic system for patients with hereditary diseases in daily clinical practice. We established a laboratory-developed test (LDT) with the implementation of whole-exome sequence analysis (WES) as the clinical sequencing system at an academic institution. WES was conducted on 81 patients with suspected hereditary disorders referred from clinical departments in our hospital from 2021 to 2024. Target genes were selected on the basis of the clinical presentations of the patients and expanded when necessary. Additional analyses, including copy number and zygosity analyses, further improved the diagnostic accuracy. The overall diagnostic yield was 42.0%, excluding four patients with transthyretin cardiac amyloidosis confirmed as wild-type ATTR Amyloidosis (ATTRwt). High diagnostic yields were associated with the presence of family histories. Secondary findings were searched in 73 patients, and pathogenic or likely pathogenic variants were disclosed to three patients, leading to their subsequent clinical follow-up. Internal and external quality controls ensured analytical reliability. This study demonstrates that the implementation of LDT-based comprehensive genome analysis is highly useful in daily clinical practice, achieving a high diagnostic yield comparable to those in previous large-scale studies; furthermore, the clinical sequencing seamlessly harmonizes the research-level investigations, providing highly valuable results. Broader insurance coverage will be essential for expanding equitable access to the genetic tests based on comprehensive genome sequence analysis.

## Introduction

With remarkable recent advances in molecular genetics research, the number of diseases for which the causative genes have been identified is increasing. As of 3 November 2025, the causative genes for 7059 diseases have been identified (https://www.omim.org/statistics/entry). Since the number of patients with individual hereditary diseases is often small and the mutational analysis of multiple genes for each disease is mandatory, comprehensive genome sequence analysis employing next-generation sequencers (NGSs) is becoming the most efficient and robust diagnostic test in clinical practice [1, 2].

In the health insurance system in Japan, however, only 193 diseases are covered by the health insurance in 2024, which is in a striking contrast to the 7059 entries as “phenotype description, molecular basis known” [3]. Thus, we need to consider how we can better provide appropriate genetic tests for diseases that are not covered by the health insurance system in Japan.

The fundamental purpose of genetic diagnosis is to apply a logical process to select candidate genes on the basis of the clinical presentations of the patients, followed by the identification of pathogenic variants responsible for the diseases. Owing to the limited coverage by the health insurance system and the limited number of genetic tests that can be provided by commercial laboratories in Japan, much of the effort is instead directed toward searching for commercial laboratories that provide the relevant tests. This situation represents a considerable deviation from the ideal patient-centered medical practice and poses a serious challenge in clinical practice.

Recent progress in molecular genetics research has also demonstrated that even for diseases showing similar clinical presentations, there are many genes responsible for the similar clinical presentations. For example, as many as 55 genes are listed as the candidate genes to establish the molecular diagnosis for patients presenting with leukoencephalopathy [4]. Thus, we need to test numerous multiple genes to establish accurate molecular diagnoses.

In Japan, the revised Medical Care Act came into effect in December 2018 to ensure the quality and accuracy of clinical laboratory tests, including genetic tests, as well as to ensure the requirements for the facilities engaged in the clinical laboratory tests. Consequently, academic laboratories are required to similarly fulfill the requirements as clinical laboratories to provide genetic tests for clinical practice, which has posed many difficulties to academic laboratories that had previously provided clinical tests. In addition, the diagnostic kits authorized for laboratory tests employing next-generation sequencers are not commercially available; hence, not only the academic laboratories but also the commercial laboratories need to develop laboratory-developed tests (LDTs) that ensure the accuracy and quality of clinical sequencing.

When whole-exome sequencing (WES) analysis on the target genes fails to yield a definitive diagnosis, complementary genetic analyses, further expanding the list of candidate genes, as well as the application of other procedures of genome analysis, may be needed to establish the molecular diagnosis. LDTs conducted in academic settings should therefore be designed to seamlessly accommodate additional research-level analyses, enabling more comprehensive diagnostic evaluation.

As a result of comprehensive genome analyses, including WES/whole-genome sequencing (WGS) analyses, pathogenic variants for diseases that are not related to the diagnostic purpose for the patients’ diseases may be obtained. Thus, from the ethical viewpoint, we need to consider the appropriate steps to return the results of secondary findings (SFs) when the patients desire disclosure of the results.

As described above, implementing a comprehensive genome analysis as a clinical sequencing method in Japan poses the following challenges. The selection of as many appropriate target genes is essential to increase the diagnostic yield. For diseases that are not covered by the health insurance system, we need to consider the appropriate steps to provide clinical sequencing. Since we occasionally face the necessity of advanced genome analysis on research levels, including analyses of structural variants, copy number variants, and determination of zygosity for multiple heterozygous variants, we need to seamlessly provide research-level analyses to accomplish a high diagnostic yield.　Accurate variant calling and interpretation of the pathogenicity of variants are essential to accomplish high-quality clinical sequencing. Quality control of clinical sequencing in accordance with the revised Medical Care Act is another mandatory issue. To address these issues, we have implemented an LDT-based clinical sequencing system in the clinical practice at our medical institution. We herein describe how we have implemented the clinical sequencing system as an LDT in our institution and discuss various issues encountered during the implementation and the practice of the clinical sequencing.

## Materials and methods

In this study, we developed and implemented a workflow to provide the best genetic tests requested by the clinical departments to establish the clinical diagnosis. The workflow is as follows: (1) comprehensive enumeration of candidate target genes relevant to the clinical presentations of patients; (2) providing the test on a self-pay basis when not covered by insurance. Tests not covered by Japanese health insurance were provided at a flat fee of 45,000 yen (49,500 yen including tax), paid by the patient or by the physician’s research funds. (3) expanding the target genes when additional genetic tests are required; (4) structured pre-test as well as post-test counseling provided by certified genetic counselors; (5) responding to the requests from patients for disclosure of SFs.

### Establishment of LDT for clinical sequencing

#### WES analysis

Genomic DNA was extracted from peripheral blood leukocytes with written informed consent. DNA libraries for WES analysis were prepared using NEBNext Ultra II FS DNA Library Prep Kit for Illumina (New England Biolabs, Ipswich, MA, USA) and xGen Exome Hybridization Panel v2 (Integrated DNA Technologies, Coralville, IA, USA). The WES analysis was conducted using NovaSeq6000 (Illumina, San Diego, CA, USA). The DNA sequences obtained were aligned to the human genome reference sequence (GRCh38/hg38) by BWA-MEM. SNVs and small indels were called using the GATK germline short variant discovery pipeline, and copy number variations (CNVs) were called using GATK-gCNV and Manta. Functional annotation was performed using VEP, referring to various databases: the Genome Aggregation Database (gnomAD; https://gnomad.broadinstitute.org), Japanese Multi Omics Reference Panel (jMorp; https://jmorp.megabank.tohoku.ac.jp), CADD (https://cadd.gs.washington.edu), SpliceAI (https://github.com/Illumina/SpliceAI), ClinVar (https://www.ncbi.nlm.nih.gov/clinvar/), and HGMD　(https://www.hgmd.cf.ac.uk/ac/index.php). The detected variants were evaluated in accordance with the guidelines for interpreting sequence variants recommended by the American College of Medical Genetics and Genomics (ACMG) and the Association for Molecular Pathology (AMP) [5], the guidelines for interpreting copy-number variants from the ACMG and Clinical Genome Resource (ClinGen), the recommendations for interpreting the loss-of-function PVS1 ACMG/AMP variant criterion from ClinGen, and the guidelines from the Association for Clinical Genomic Science (ACGS).

#### Selection of target genes for WES

We conducted clinical sequencing after receiving the requests from the clinical departments. They provided the clinical diagnosis, including the candidate genes that are directly relevant to the clinical diagnosis (genes in list A). When other diseases with clinical presentations may partly overlap with the clinical diagnosis of the patient, target genes were added at the Center for Genetic Diagnosis to cover the diseases with an expanded disease spectrum relevant to the clinical presentations of the patient (genes in list B). To accomplish this, we searched OMIM and PubMed to pick up diseases that overlap with the clinical presentation of the referred patient. When pathogenic variants were not identified in the genes in lists A or B, we re-evaluated the clinical presentations of the patients and considered the possibility of further expanding the gene lists (genes in list C). The algorithm of selection of target genes is shown in Fig. 2 and Supplementary Table 1.

#### Genes for SFs

Before conducting the clinical sequencing, we explained to the patients the genes listed as SFs, which include 81 genes that are recommended by the American College of Medical Genetics and Genomics (ACMG SF v3.0, v3.1, v3.2) [6–8] as the genes useful for the health care of the patients. We ask the patients whether they opt for the disclosure of the results when pathogenic/likely pathogenic variants are identified. The pathogenic/likely pathogenic variants were explained to the patients by clinical geneticists and certified genetic counselors, followed by referring them to the clinical departments specialized in the relevant diseases for follow-up.

#### Quality control

Internal quality control was performed using standard genomic DNAs recommended by the Genome in a Bottle (GIAB) Consortium, organized by the National Institute of Standards and Technology (NIST) [https://www.nist.gov/programs-projects/genome-bottle]. The genomic DNAs were processed following the same protocols that were used for the WES analysis of the patients’ genomic DNAs. The target regions for WES, excluding difficult-to-interpret regions defined by GIAB, were evaluated by comparing the called variants with the reference set of 16,523 confirmed variants. Precision and recall were calculated and used as indicators of analytical quality. In addition, as part of external quality control, our laboratory participates in the College of American Pathologists (CAP) proficiency tests program [https://www.cap.org].

#### IRB approval

This study was approved by the Institutional Review Board of the International University of Health and Welfare (Research on clinical sequencing of hereditary diseases using comprehensive genome analysis. 21-Im-007-2).

#### Registration as an authorized clinical laboratory (“hygiene testing laboratory”)

In accordance with the revised Medical Care Act, our facility was registered as an authorized clinical laboratory (hygiene testing laboratory) in 2021.

#### Procedures of clinical sequencing in clinical practice

**Fig. 1:**
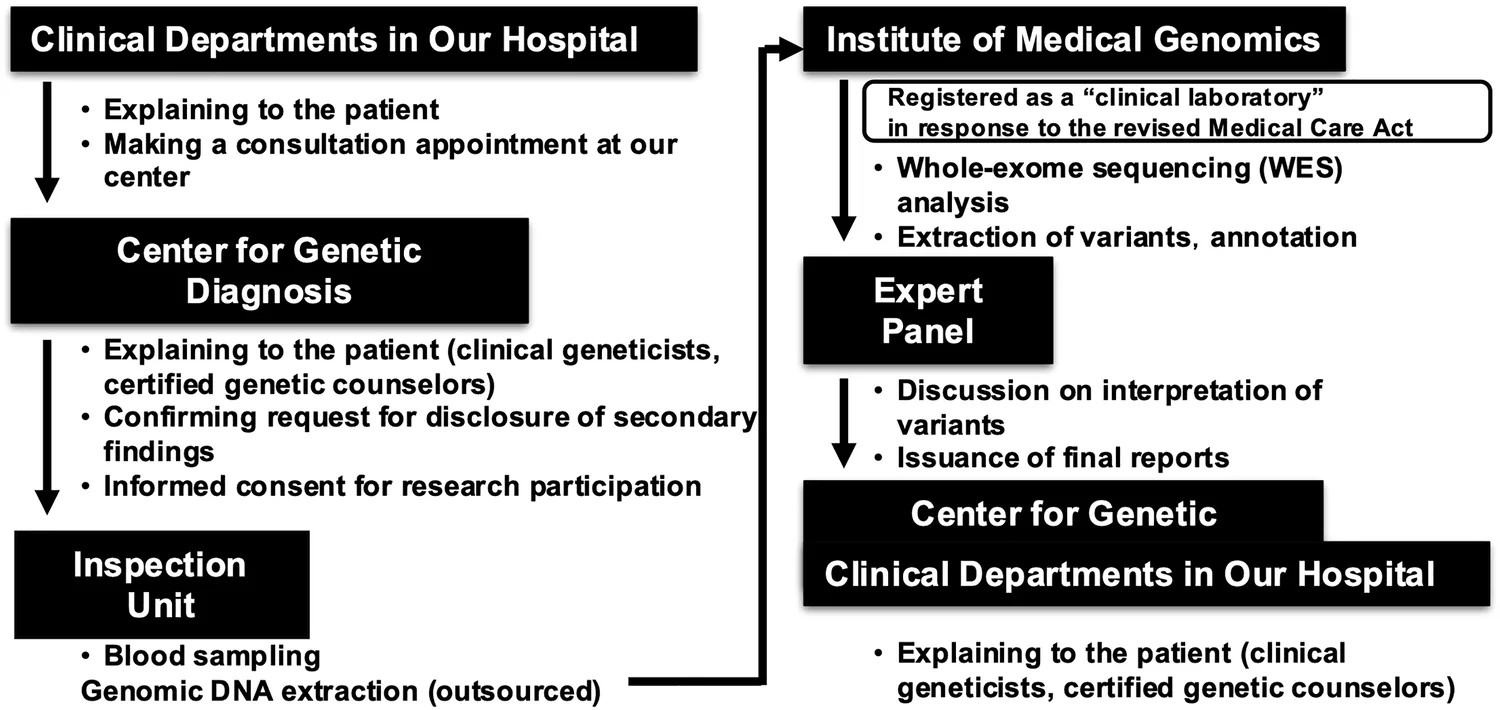
Flowchart of clinical sequencing at our hospital

The framework of the clinical sequencing is described in Fig. 1. We provide clinical sequencing services upon request from the clinical departments in our hospital. The clinical departments provide the clinical diagnosis and the gene(s) required to support the clinical diagnosis. The patients then visited the Center for Genetic Diagnosis. Prior to the genetic tests, patients are fully informed about the disease and the genetic tests. Genetic counseling is provided by clinical geneticists and certified genetic counselors. Depending on the clinical presentations and the family history, we optionally add target genes to be analyzed to accomplish high diagnostic yields. As the representative cases, 37 genes are selected for analysis of HCM, 16 genes for long QT syndrome, 18 genes for autoinflammatory diseases, 32 genes for congenital connective tissue disorders, 21 genes for muscular disorders, and 21 genes for salt-losing tubulopathy (Supplementary Table 1). When no pathogenic variants are identified, additional analyses are performed, including reconsideration of candidate genes and evaluation of CNVs. We conduct additional tests to confirm homozygosity in diseases with autosomal recessive inheritance, or to confirm zygosity in the case of multiple heterozygous variants in diseases with autosomal recessive inheritance, as needed to assess the pathogenicity of the variant.

In addition, the procedure for the disclosure of any secondary findings (SF) is explained to the patients in advance. Written informed consent is obtained from all the patients prior to the genetic tests.

After discussing the interpretation of the identified variants in the in-house expert panel, we issue reports and disclose the results to the patients. The expert panel includes the attending physicians who requested the test, the members of the Center for Genetic Diagnosis, and the clinical sequencing committee. The turnaround time (TAT) is set at about three to four months. The final report is registered in the electronic medical record in our hospital. Access to the reports is limited to authorized medical staff.

Disclosure of the results of the clinical sequencing is made in person on the basis of the written report of the clinical sequencing. When a patient requests the disclosure of the pathogenic variants identified in the list of genes for the secondary findings, the results are disclosed to patients along with the written reports. The disclosure is made in person with the clinical geneticists and certified genetic counselors in attendance.

### Genetic counseling

Genetic counseling is provided by Certified Genetic Counselors. Written informed consent was obtained from all the patients prior to the genetic tests. Genetic counseling was also offered to the patients and to the family members when considered appropriate at the time of disclosure of results. For pediatric patients, written informed consent was obtained from the parent, and informed assent was sought from the minors judged capable after age-appropriate explanation. Secondary findings were managed according to ACMG SF v3.2 [8]; in the minors, disclosure was made in the child’s best interests in consultation with parents.

## Results

### Demographic characteristics of patients

The demographic characteristics of the patients who underwent the clinical sequencing are summarized in Table 1. A total of 81 patients suspected of having hereditary disorders were referred to the Center for Genetic Diagnosis from the 13 clinical departments (Cardiology, Ophthalmology, Dermatology, Allergy and Clinical Immunology, Oncology, Nephrology, Hematology, Vascular Surgery, Neurology, Diabetes, Metabolism and Endocrinology, Cardiac Surgery, Otorhinolaryngology, and Thoracic Surgery) in our hospital from 2021 to 2024.

**Table 1:** Demographic Characteristics of Patients

Sex		
	Male	37 (45.7%)
	Female	44 (54.3%)
Median age (y)		53 (0–83)
Clinical departments		
	Cardiology	26 (32.1%)
	Ophthalmology	20 (24.7%)
	Dermatology	7 (8.6%)
	Allergy and Rheumatology	6 (7.4%)
	Oncology	4 (4.9%)
	Nephrology	4 (4.9%)
	Hematology	3 (3.7%)
	Vascular Surgery	3 (3.7%)
	Neurology	3 (3.7%)
	Diabetes, Metabolism and Endocrinology	2 (2.5%)
	Cardiac Surgery	1 (1.2%)
	Otorhinolaryngology	1 (1.2%)
	Thoracic Surgery	1 (1.2%)
	Total	81
Insurance Coverage	Yes	68 (84.0%)
	No	13 (16.0%)
Request for disclosure of secondary findings	Yes	73 (90.1%)
	No	8 (9.9%)

### Workflow of WES-based clinical sequencing and diagnostic yield

**Fig. 2:**
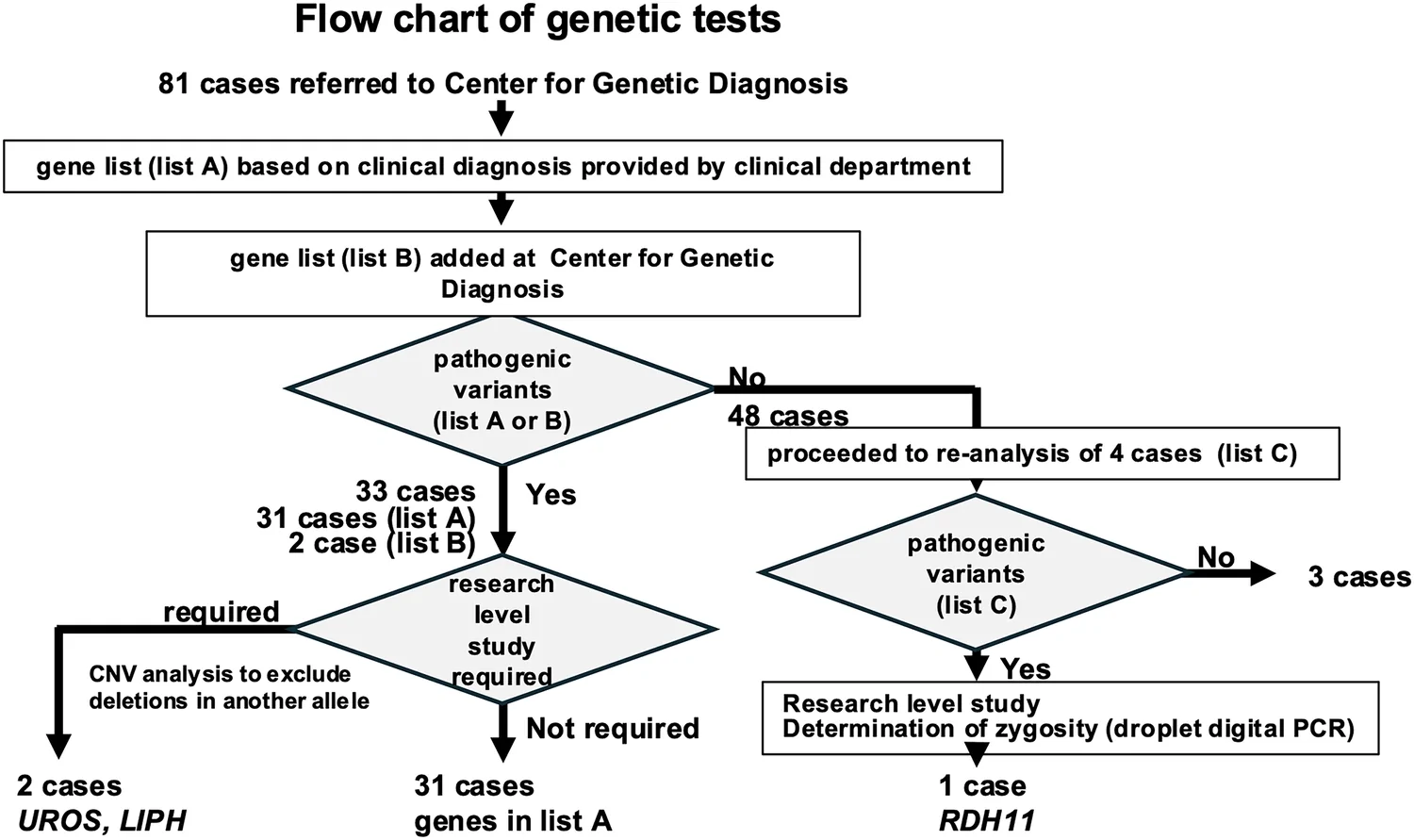
Flow chart of genetic tests at our facility

The workflow and outcomes of the clinical sequencing are summarized in Fig. 2. In the workflow, pathogenic or likely pathogenic variants were identified in 31 patients in list A, two patients (*UROS, DHDDS*) in genes in list B and one patient (*RDH11*) in list C (Fig. 2 and Supplementary Table 1).

The candidate variant was identified in *UROS* in list B. Although the initial clinical diagnosis was “porphyria cutanea tarda”, for which *UROD* is the causative gene, pathogenic variants were not detected in *UROS*. We then expanded the candidate genes to include *UROS*, *ALAD*, *PPOX*, *CPOX*, *ALAS2*, *FECH*, and *HMBS* (list B). As a result, we identified an apparently homozygous likely pathogenic variant in *UROS*. CNV analysis based on the WES data excluded the possibility of a deletion in another allele, which confirmed the diagnosis of congenital erythropoietic porphyria (CEP) (Fig. 2). The patient is a 66-year-old male with onset of skin erosions at age 66, an atypical case of late adulthood onset CEP. As an example of the gene in list C, the initial clinical diagnosis of the patient was “progressive muscular dystrophy”. Pathogenic variants were neither found in the 28 genes responsible for progressive muscular dystrophy (list A) nor in the 115 additional genes selected for diseases presenting with myopathy as the main clinical presentation (list B). We then conducted a comprehensive search for pathogenic variants using the whole WES data, and found two rare heterozygous variants in *RDH11*. Droplet digital PCR analysis confirmed compound heterozygosity of the variants, thus confirming the diagnosis of retinitis pigmentosa associated with myopathy (Supplementary Table 1) [9].

**Fig. 3:**
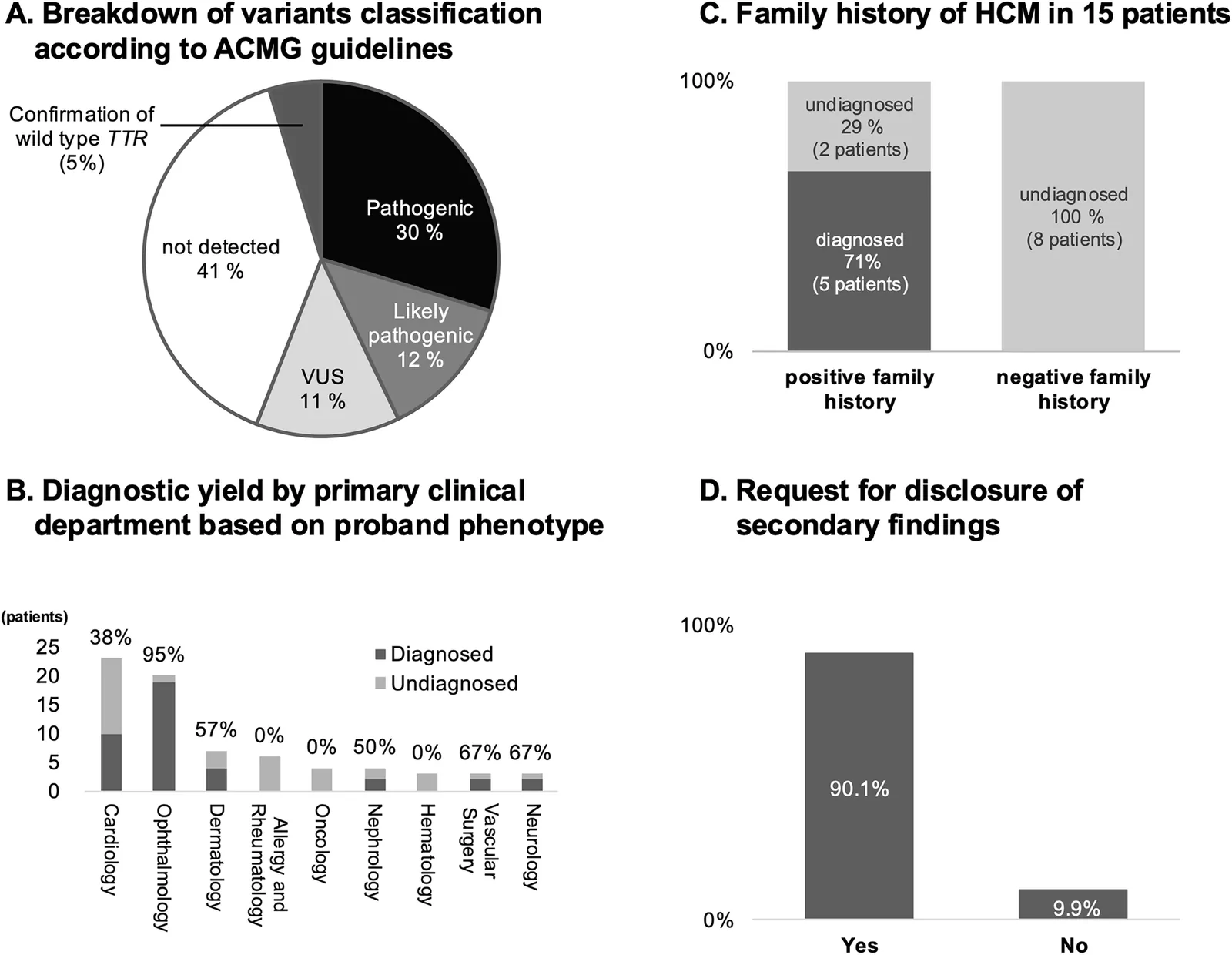
Results of clinical sequencing analysis, including variant classification, diagnostic yield, family history, and request for disclosure of secondary findings. **A** Breakdown of classification of variants identified in the 81 patients who underwent clinical sequencing. Molecular diagnosis was established in 34 patients, excluding four patients with transthyretin cardiac amyloidosis confirmed as wild-type ATTR Amyloidosis (ATTRwt). Since biallelic variants were identified in diseases with autosomal recessive inheritance, the total number of pathogenic or likely pathogenic variants was 35. **B** Diagnostic yields per clinical departments. **C** Family history of HCM in 15 patients. Among the seven patients with positive family histories, definitive genetic diagnosis was made in five patients (71.4%), whereas none of the eight patients with negative family histories were genetically diagnosed. **D** Proportion of patients who request disclosure of SFs. Of the 81 patients who underwent the clinical sequencing, 73 (90.1%) requested analysis of SFs and disclosure of the results when pathogenic variants were identified

As described above, we identified pathogenic or likely pathogenic variants, including seven novel pathogenic or likely pathogenic variants in 34 of the 81 patients, resulting in the diagnostic yield of 42.0% (Fig. 3A, and Supplementary Table 1). In the 34 patients with pathogenic or likely pathogenic variants, there were 19 independent variants, which included 11 missense, five frameshift, two splice, one in-frame, and one nonsense variant. Among the 81 patients, four with transthyretin cardiac amyloidosis (ATTR-CA) were referred to the Center for Genetic Diagnosis to exclude the possibility of variant transthyretin amyloidosis (ATTRv) caused by pathogenic variants of *TTR*. Consequently, all the patients were found to carry wild-type *TTR*, which confirmed the diagnosis of wild-type transthyretin amyloidosis (ATTRwt). When the four patients with ATTRwt were included, the diagnostic yield of the clinical sequencing was 46.9%.

The diagnostic yields substantially varied depending on the clinical diagnoses, with the highest diagnostic yield of 95% observed in patients with ophthalmic diseases, primarily corneal dystrophies (Fig. 3B). Among the seven patients with HCM with positive family histories consistent with the autosomal dominant mode of inheritance, five patients (71.4%) carried pathogenic or likely pathogenic variants responsible for HCM, whereas none of the patients with a negative family history carried any pathogenic or likely pathogenic variants (Fig. 3C).

### Variants of uncertain significance (VUS)

There were 12 variants of uncertain significance (VUS). Of these, four were disease-related, whereas the remaining eight variants were not directly related to the diseases of the patients (Supplementary Table 2). To establish the pathogenicity of the four disease-related variants, further investigations are required, including confirmation of de novo variants, aberrant splicing, functional analysis, and zygosity confirmation.

### Secondary findings (SFs)

Of the 81 patients analyzed, 73 patients (90.1%) requested SF analysis, of whom pathogenic or likely variants were identified and disclosed to three patients, leading to their subsequent clinical follow-up (Fig. 3D and Table 2). A variant, c.4162 C > T (p.Arg1388Cys), in *FBN1* was identified in a patient. Although this variant was classified as a VUS according to the ACMG guidelines, it represents a cysteine-creating substitution. After review by a Marfan syndrome specialist, the variant was interpreted as likely pathogenic. Given the potential risk of nonsyndromic aortic dissection, the findings were disclosed to the patient, who subsequently underwent follow-up examinations at the Department of Cardiovascular Medicine.

**Table 2:** List of variants disclosing secondary findings

Age at testing	Gender	Clinical diagnosis	Family history of SF	Gene	Coordinates (hg38) Variant (HGVS)	ACMG variant classification	ACMG evidence	diagnosis of SF	Inheri-tance	clinical management for SF
36	F	Fabry disease	ー	*FBN1*	chr15: 48766500 G > A c.4162 C > T(p.Arg1388Cys)	VUS	PP3, PS4_ supporting	Marfan syndrome	AD	Regular aortic imaging test
46	F	Long-QT syndrome	+	*BRCA1*	chr17: 43091891 G > T c.3640 G > T(p.Glu1214Ter)	Pathogenic	PVS1, PS1, PM2, PP3	HBOC	AD	Managed by the oncology department
8 m	M	Congenital ichthyosis	ー	*APOB*	chr2: 21006289 G > A c.10579 C > T(p.Arg3527Trp)	Likely pathogenic	PS1, PM5, PP3	Familial hyper- cholesterolemia	AD	Managed by pediatrics department

*AD* autosomal dominant, *HBOC* hereditary breast and ovarian cancer, *SF* secondary findings, *VUS* variant of uncertain significance

## Discussion

### Clinical sequencing conducted at an academic institution

Here, we report the results of the clinical sequencing carried out in our institution, including 81 patients with suspected hereditary diseases who underwent clinical sequencing over a 3-year period. WES analysis was conducted as a genetic test on the 81 consecutively enrolled patients. The overall diagnostic yield was 42.0% (46.9% when four patients with ATTRwt were included), which is comparable to previously reported diagnostic yields [1, 2]. Of the 34 pathogenic or likely pathogenic variants identified in this study, seven were novel variants that had not been previously reported, which expands the mutational spectrum of the corresponding diseases and highlights the contribution of the present clinical sequencing in identifying clinically relevant variants that may be underrepresented in existing databases. In the workflow of the current clinical sequencing (Fig. 2), we flexibly expanded the gene list, which includes not olnly the genes directly related to the clinical diagnosis proposed by each clinical department (**genes in list A**), but also the genes expanded on the basis of the clinical presentations of the patients and family history, such that a broad range of phenotypically plausible diseases were included for the analysis (**genes in list B**), and re-analysis of the WES data (**genes in list C)**. Pathogenic and likely pathogenic variants were identified in *UROS* and *DHDDS* (**list B)**, and in *RDH11* **(list C)**, demonstrating the role of the workflow in the WES-based clinical sequencing (Fig. 2 and Supplementary Table 1). Although it is not easy to narrow down to the causative variants among an enormous number of rare variants identified in WES, this unbiased, comprehensive approach is certainly an advantage of WES. Thus, a comprehensive analysis of flexibly selected candidate genes is a huge advantage of WES. Furthermore, as another advantage of WES, reanalysis of the additional candidate genes, and ultimately the whole WES data, can easily be conducted. To date, there have been no reports in Japan of comprehensive genomic analyses conducted in a prospective manner based on the requests from all the clinical departments within a hospital.

The diagnostic yield considerably varied depending on the diseases. Among the patients analyzed, hypertrophic cardiomyopathy (HCM) and corneal dystrophy (CD) were the two most frequently referred diseases. These diseases exhibited distinct patterns in the relationship between the family history and the diagnostic yield. In HCM with documented family history, the diagnostic yield was as high as 71.4%, reflecting the high penetrance of the pathogenic variants in the known causative genes for HCM (Fig. 3C). When family history was absent, no pathogenic variants were identified. Although the number of patients is limited, the results suggest that family history is important for considering the possibility of HCM. The high diagnostic yield of CD is remarkable. Of note, the pathogenic variants were suggested by the ophthalmologists, indicating a strong genotype–phenotype correlation in CD. Of the 19 CD patients in whom pathogenic variants were identified, 11 (57.9%) had no clearly documented family histories. Previous reports have suggested that the high frequency of undocumented family histories in patients confirmed to have CD may be attributable to incomplete penetrance [10]. Moreover, although CD is inherited as the autosomal dominant mode of inheritance, its late onset may reduce the likelihood that affected individuals within the same family are recognized as having similar symptoms. Inclusion of patients with CD in the present study certainly contributed to the increased diagnostic yield.

Among the 33 patients (40.7%) in whom no causative variants were identified, eight patients had similar cases within the family, suggesting a genetic etiology, whereas the remaining 25 patients had no clear family history and the clinical presenttions were atypical; hence, an association with a genetic disease was not strongly suggested (Fig. 3A). Our experience emphasizes that not only clinical evaluation but also insights into the genetic background of patients based on information of the family history are helpful.

### Secondary findings

The high proportion of patients (90.1%) opting to receive disclosure of SFs underscores the importance of clear pretest counseling. In this study, pathogenic or likely pathogenic variants identified in three patients led to disease-specific follow-up by appropriate specialists, supporting the clinical utility of SF disclosure for proactive care. Given the ethical complexities of unsolicited genomic information, ensuring informed and autonomous decision-making is essential. Further standardization in the reporting of SFs will be essential as genomic tests become more widely available in clinical practice. When addressing SFs in pediatric settings, it is essential to assess each case individually for assent, carefully considering the pediatric patients’ capacity for decision-making as well as the consent from the parents, and the potential clinical benefits of early disclosure of such SFs.

### The role of genetic counselors

In our clinical sequencing program, certified genetic counselors were involved in both pre- and post-test counseling, playing a critical role in facilitating communication with the patients and providing sufficient information about the genetic test, as well as in the appropriate handling of secondary findings.

### Long TAT

A TAT of 3–4 months is relatively long for a routine clinical test. However, when the primary objective is to maximize diagnostic yield, a longer TAT is often unavoidable. Several factors contribute to the prolonged TAT in our clinical sequencing workflow. First, samples are analyzed in batches after enough specimens have been accumulated for WES. Second, as the number of target genes increases, additional time is required for the appropriate interpretation of variants. Third, the analyses may occasionally be performed at the research level, such as assessment of CNVs and zygosity, which resulted in additional time. Fourth, when no pathogenic variants are initially identified in genes in lists A or B, reanalysis of the WES data is conducted (list C), including genes for a broader spectrum of related diseases, and even for the entire WES data; this process substantially increases the total time required for the reanalysis. Fifth, analysis of 81 genes for SF further required additional time. Finally, in cases in which no pathogenic variants are detected, determining the appropriate endpoint of analysis is inherently challenging. Whereas identification of a pathogenic/likely pathogenic variant allows the analysis to conclude, negative results frequently necessitate further extensive evaluation and discussion. Thus, there was an inherent trade-off between TAT and the extent of the genetic analysis, and we consider efforts to maximize diagnostic yield to be of clinical significance. When rapid results were clinically required, we communicated directly with the attending physicians and provided expedited reporting whenever possible.

### Health insurance issues in Japan

In this study, WES was performed for 33 diseases, of which 19 (57.6%) were covered by the public health insurance in Japan, whereas 14 (42.4%) required payment by the patients themselves. This financial burden may lead to some patients declining the genetic tests, highlighting the need to expand insurance coverage to ensure equitable access to diagnosis. It should be emphasized that the establishment of the diagnosis is by far the most essential point in clinical practice, regardless of the availability of highly efficacious treatment. Moreover, comprehensive WES/WGS can serve as a one-time test, with genomic data reusable for future clinical care, thereby maximizing long-term patient benefit.

### Role of academic laboratories in clinical sequencing

In clinical sequencing based on WES, the selection of the candidate genes can be flexibly customized in accordance with the clinical presentations of the patients, and re-analysis can be performed retrospectively to improve the diagnostic yield [2, 11]. In contrast, commercially available genetic tests typically limit analysis to a predefined gene panel that includes only a limited number of genes, and further investigation is usually discontinued when no conclusive results are obtained. Given the increasing recognition of genetic heterogeneity and overlapping phenotypes, reliance solely on the prefixed gene lists is of limited role in achieving a definitive diagnosis in many cases. Flexible inclusion of additional genes in the initial target gene list is expected to increase the diagnostic yield.

Currently, the clinical sequencing based on comprehensive genome sequence analysis is positioned somewhere between the tests conducted at commercial laboratories and those conducted at academic laboratories. Since the diagnostic kits employing next-generation sequencers approved by the Act on Securing Quality, Efficacy and Safety of Products Including Pharmaceuticals and Medical Devices (PMD Act) are not available, the genetic testing is not conducted by automated machines routinely at commercial laboratories. Since the diagnostic rate is around 40% as we demonstrated, additional research-level studies, including determination of zygosity, copy number analysis and reanalysis of the WES data, are occasionally required. These additional tests cannot be completed at commercial laboratories. All these situations strongly argue for the necessity of LDT conducted at academic laboratories.

### Final issues at academic laboratories

The expenses associated with LDT-based sequencing include additional analyses such as CNV analysis. Confirmation of zygosity and reanalysis of the WES data, as well as those for operation and maintenance of the laboratory, are managed within a research framework, for which informed consent is obtained under protocols approved by the institutional research ethics committee. These expenses are supported by research funding. For the maintenance of machines that are approved by the PMD Act, a certain amount of expenses is mandatory to regularly check the performance of the machines. It is not easy to cover such expenses in academic laboratories. Thus, financial support from the research fundings would be required. But such funding is not easy to obtain. Since the diagnostic yield is around 40%, we should recognize that research activities are absolutely required to advance molecular genetics research and genetic testing. Although this may be difficult to accomplish, it would be desirable that suitable funding systems be made available to the academic laboratories that undertake clinical sequencing, along with molecular genetic research.

### Considerations regarding the revised Medical Care Act

Our laboratory was registered as an authorized clinical laboratory (hygiene testing laboratory) in 2021 to comply with the revised regulatory act, which requires significant effort in managing laboratory facilities, securing maintenance costs, and employing specialized staff. Although the regulation is appropriate for the management of commercial laboratories that undertake an enormous number of laboratory tests, it may not be suitable for quality management in academic laboratories that conduct a limited number of tests among a broad range of laboratory tests, with each requiring expert skills and experienced personnel. Moreover, many academic laboratories have stopped laboratory tests owing to the burden of complying with the act. These movements may discourage the initiatives of academic laboratories, leading to the undesirable stalling of genetic research, which should be fully acknowledged.

Regarding the assurance of quality control of the genetic testing conducted at academic laboratories, it should be emphasized that academic laboratories usually perform genetic testing that is related to the research activities conducted at their own academic laboratories. Therefore, individual laboratories can set up appropriate quality control measures of the genetic testing that is optimized for those conducted at their own academic laboratories.

### Limitation

Owing to the technical limitations of WES, there may be cases that remain undiagnosed by WES. Variant types such as CNVs, tandem repeat expansions, and deep intronic variants are often difficult to detect by WES. WGS, in contrast, offers several potential advantages, including the capability to detect CNVs and deep intronic variants that WES may miss. However, WGS substantially increases analytical burden and costs. Existing reports indicate that the incremental diagnostic advantage of WGS over WES is relatively small and may not currently support its widespread clinical use [12–17]. Detection of expanded tandem repeats by WGS employing currently popular short-read sequencers is difficult. Given the increasing number of identified genes with expanded tandem repeats, the application of long-read sequencers is highly useful for the comprehensive analysis of such expanded tandem repeats. Comprehensive genome sequence analysis employing long-read sequencers, however, is still expensive to incorporate into clinical sequencing.

## Supplementary information

Supplementary Table 1

Supplementary Table 2
